# Methylation-specific, universal, and highly sensitive identification of ctDNA in prostate cancer by multiplex droplet digital PCR

**DOI:** 10.1371/journal.pone.0340786

**Published:** 2026-01-29

**Authors:** Søren Kahns, Ahmed H. Zedan, Mads M. Aagaard, Luisa M. D. Canto, Sanne Kjær-Frifeldt, Stine V. Eriksen, Christine V. Madsen, Jonna S. Madsen, Palle J. S. Osther, Torben F. Hansen, Rikke F. Andersen

**Affiliations:** 1 Department of Biochemistry and Immunology, Vejle Hospital, University Hospital of Southern Denmark, Vejle, Denmark; 2 Department of Oncology, Vejle Hospital, University Hospital of Southern Denmark, Vejle, Denmark; 3 Department of Genetics, Vejle Hospital, University Hospital of Southern Denmark, Vejle, Denmark; 4 Department of Pathology, Vejle Hospital, University Hospital of Southern Denmark, Vejle, Denmark; 5 Department of Regional Health Research, Faculty of Health Sciences, University of Southern Denmark, Odense, Denmark; 6 Department of Urology, Vejle Hospital, University Hospital of Southern Denmark, Vejle, Denmark; The University of Texas MD Anderson Cancer Center, UNITED STATES OF AMERICA

## Abstract

**Objective:**

Liquid biopsy for detection of circulating tumor DNA (ctDNA) offers a promising, non-invasive strategy for real-time monitoring of cancer. We aimed to develop a universal and sensitive one-tube droplet digital PCR (ddPCR) multiplex assay able to detect ctDNA in plasma from metastatic prostate cancer (PCa) patients.

**Methods:**

Publicly available array data are applied to identify methylation signatures specific to both normal and cancerous prostate tissue. The assay is based on the highly sensitive ddPCR technology, and tissue and plasma samples are analyzed using the QX600 ddPCR multiplexing platform from BioRad.

**Results:**

We identified three novel genomic regions, *ACTRT2*, *EVX1,* and *HOXD13*, containing CpG-methylation signatures specific to normal and cancerous prostate tissue. We successfully targeted the three biomarkers in a single-tube methylation-specific multiplex ddPCR (mm-ddPCR) assay along with two previously reported PCa-specific hypermethylated CpG biomarkers, *DOCK2* and *HAPLN3*, and the *ALB* reference gene. The mm-ddPCR assay consistently detected *ACTRT2*, *EVX1,* and *HOXD13* across all samples originating from prostate-derived tissue. In contrast, the *HAPLN3* and *DOCK2* biomarkers were predominantly detected in PCa tissue. We also demonstrate that the five ctDNA biomarkers can be detected in plasma samples from metastatic castration-resistant PCa (mCRPC) patients with a 95% CI (85%, 100%) using the mm-ddPCR assay.

**Conclusion:**

This work demonstrates that the mm-ddPCR assay offers a simple, fast, cost-effective, sensitive, and universal tool for the detection of ctDNA biomarkers in plasma from mCRPC patients. Further studies are required to explore its potential for PCa risk stratification and treatment guidance.

## Introduction

A significant challenge in prostate cancer (PCa) management is distinguishing between non-aggressive and aggressive disease to avoid overdiagnosis and overtreatment [1–3]. Prostate-specific antigen (PSA) in plasma is the primary biomarker for PCa screening and monitoring [4], but due to its low specificity, PSA cannot reliably discriminate between benign and malignant disease [5,6]. Despite advances in diagnostic and staging modalities such as magnetic resonance imaging (MRI) and prostate-specific membrane antigen (PSMA) positron emission tomography, the identification of patients who would benefit from intensive treatment and the establishment of effective monitoring strategies remain critical challenges. This underscores the need for better and more reliable biomarkers in PCa management.

Circulating tumor DNA (ctDNA) is a promising biomarker for close to real-time monitoring of cancer treatment by liquid biopsies [7,8]. In cancer patients, ctDNA is shed from tumor cells into the blood. It has, however, been suggested that due to neoplastic growth DNA is also shed from normal epithelial cells adjacent to cancer cells [9]. In patients with early-stage cancer, e.g., localized disease, the ctDNA levels are often low whereas they can generally be detected in patients with a high tumor burden [10,11]. In metastatic castration-resistant prostate cancer (mCRPC), a high fraction of ctDNA has been associated with a poor prognosis [12–14]. Monitoring the fraction of ctDNA during mCRPC treatment has been reported as a potentially valuable tool for risk stratification and treatment guidance with ctDNA performing superior to or complementing PSA measurements [15,16]. A recent report showed that the fraction of ctDNA is a measure of when it is clinically relevant to use ctDNA for cancer genotyping and identification of actionable mutations [13].

The technological platforms used to detect ctDNA biomarkers in liquid biopsies are primarily digital polymerase chain reaction (dPCR) and next-generation sequencing (NGS) [17]. NGS provides sequence information on all ctDNA fragments detected in a sample and is generally expensive to run and requires extensive analysis time. Technologies based on dPCR are highly sensitive, faster, and more cost-effective than NGS, but they require prior knowledge of the target [18]. Cancer cells exhibit aberrant epigenetic patterns, for instance in Cytosine-phosphate-Guanine dinucleotide (CpG)-methylation patterns on certain genes. It has been reported that the methylation pattern provides a universal biomarker with low inter-individual variability and high tumor specificity and sensitivity [9,19,20]. The ctDNA methylation landscape in cancer patients includes a mixture of tissue-conserved and cancer-derived methylation profiles all originating from the diseased organ [10,21]. Targeting tissue-conserved methylation markers preserved in the tumor tissue has been reported to increase sensitivity [9].

The objective of this study was to develop and validate a novel single tube, methylation-specific multiplex ddPCR (mm-ddPCR) tool for sensitive, universal, low-cost and high-throughput detection of ctDNA biomarkers in plasma from PCa patients. Studies indicate that targeting multiple biomarkers enhance the sensitivity of ctDNA detection [19,22]. Our multiplex assay targets five methylation-specific ctDNA biomarkers and a reference gene in a single tube. We identify and target three genomic regions, *ACTRT2*, *EVX1,* and *HOXD13,* where CpG-methylation patterns are conserved in cancerous as well as normal prostate tissue. ctDNA biomarkers, *HAPLN3* and *DOCK2*, have previously been described as hypermethylated in PCa tissue [11]. The combination of tissue-conserved and cancer-specific markers are thought to increase the sensitivity and specificity of the test. This strategy can also be instrumental in identifying shedding differences between the two biomarker types. For simplicity, we refer to all five targeted biomarkers as ctDNA, assuming that these circulating cell-free DNAs (ccfDNA) originate from either PCa cells or prostate cells affected by cancer.

## Materials and methods

### Biomarker discovery using Illumina 450K methylation array data

Infinium® Human Methylation450 BeadChip array data from The Cancer Genome Atlas (TCGA) were applied. Data on normal prostate (PNo) tissue adjacent to cancer cells from the prostate (PNo, n = 50), other available tissue types (n = 695), and PCa tissues (n = 503) were downloaded from the Genomic Data Commons (GDC) data portal in April 2020 (S1 Table) [23]. In addition, a selection of previously published and publicly available datasets was incorporated: GSE67393 (peripheral blood, n = 117) and GSE121192 (peripheral blood monocytes, n = 16) [24]. The total dataset is based on 1381 samples, i.e., 553 prostate (50 PNo and 503 PCa) and 828 non-prostate benign samples (tissue and blood).

All data analyses were performed in R (v. 4.0.5) using the packages caret (v.6.0–92), randomForest (v. 4.6–14), mlbench (v. 2.1–3), pheatmap (v. 1.0.12), ggplot2 (v.3.3.5), dplyr (v. 1.0.7), readr (v.2.1.2), and minfi in addition to functions in base packages [25].

Methylation probes with missing values were filtered out. Mean beta values were then calculated for all prostate samples (PRAD, n = 553) and all non-prostate samples (REST, n = 828). An initial list of differentially methylated CpGs (DMC) was extracted by selecting sites with mean PRAD beta > 0.75 and mean REST beta <0.25 (PRAD hypermethylated sites) OR mean PRAD beta <0.25 and mean REST beta >0.75 (PRAD hypomethylated sites). Only sites mapping to CpG islands were included (S2 Table). Next, recursive feature elimination (RFE) was employed using 10-fold cross-validation (repeated five times) to identify the DMCs best suited for separating PRAD from REST samples. CpG islands containing more than one unique DMC were selected by manual inspection of the beta value distribution for each sample group, i.e., each TCGA tissue type or GSE dataset (S3 Table).

### Ethics statements

Samples were obtained from the ongoing PerPros Biobank (enrollment start 15.09.2015; data accessed 18.08.2022) and the biobank of the protocol “Biomarkers in patients with symptomatic, metastatic hormone-refractory prostate cancer during docetaxel treatment (enrollment 29.05-2012-10.10.2018; data accessed 04.12.2023)”, Department of Oncology, Vejle Hospital, Denmark. The biobanks are registered with the Region of Southern Denmark (18/11174 and 18/3908). This study was approved by The Regional Committees on Health Research Ethics for Southern Denmark (S-20240017 and S-20110102). Patient data used in this study were pseudonymized with no access to information that could identify patients during or after data collection. All participants provided written and orally informed consent, which was saved in the Research Electronic Data Capture (REDCap) tool hosted by the Open Patient Data Explorative Network (OPEN), Denmark. The principles outlined in the Declaration of Helsinki were followed throughout the study. We confirm that all methods were performed in accordance with relevant guidelines and regulations.

### In-house samples

Formalin-fixed, paraffin-embedded (FFPE) histologically verified prostate tissue specimens (PNo n = 21 and PCa n = 20) were collected together with colorectal (n = 5), lung (n = 5), breast (n = 5), and ovarian (n = 5) tissue. Whole blood was obtained from 20 men not examined for nor diagnosed with PCa as well as plasma from 80 men examined for but not diagnosed with PCa. Baseline plasma sampled before the first cycle of docetaxel was obtained from 20 men diagnosed with mCRPC.

### Extraction of DNA from tissue, whole blood, and plasma

DNA was extracted from FFPE biopsy specimens on the Maxwell RSC instrument using the Maxwell RSC FFPE Plus DNA Kit (Promega, Madison, WI, USA) according to the manufacturer’s instructions. Briefly, FFPE tissue sections were incubated in 180 µl incubation buffer and added 20 µl of a 20 mg/ml Proteinase K solution at 70°C overnight. 400 µl lysis buffer was added before the DNA was extracted according to the manufacturer’s instructions. The extracted DNA was eluted in 50 µl nuclease-free water.

DNA from whole blood was extracted from 200 µl on the QIAsymphony SP instrument using the QiaSymphony DSP DNA Mini Kit (Qiagen, Hilden, Germany) according to the manufacturer’s instructions. Extracted DNA was eluted in 400 µl elution buffer.

Plasma was isolated by centrifugation of blood at 2,000g for 10 minutes within 4 hours of collection in EDTA tubes and subsequently stored at −80°C. Before DNA purification, plasma was centrifuged at 10,000g and added 35,000 copies per ml plasma of the exogenous *CPP1* for process control [26]. DNA was extracted from either 2 ml or 4 ml of plasma on the QIAsymphony SP instrument using the QIAsymphony DSP circulating kit (Qiagen, Hilden, Germany) according to the manufacturer’s instructions. DNA was eluted in 60 µl elution buffer and added 140 µl H_2_O. Thirty µl was used for quality control (QC), with ddPCRs for *CPP1* and *PB* analyzed on the QX200 system (Bio-Rad) as QC for purification efficiency and contamination level of peripheral blood cell DNA in the samples, respectively [26]. In addition, extracted DNA fragmentation length was controlled by ddPCR quantification of 65 bp and 250 bp fragments of the *ECM7* gene [27].

### Bisulfite conversion of DNA

A total of 170 µl purified DNA was concentrated to 20 µl in Amicon Ultra 30K 0.5 ml columns and subsequently bisulfite converted using the EZ methylation-Lightning kit (Zymo Research, Irvine, CA, USA) as described by the manufacturer. Bisulfite-converted DNA was eluted in 15 µl elution buffer. Universal methylated and unmethylated Human DNA Standards (BioSite-D5014, Zymo Research) were bisulfite converted with the samples and used as positive/negative controls as well as bisulfite conversion controls. The recovery of DNA after bisulfite conversion was estimated for each sample as the ratio between the concentration of the converted *ALB* reference gene and the concentration of non-converted *ECM7* 65 [27]. Acceptance criteria were set at a ratio>0.2.

### Design and optimization of methylation-specific ddPCR assays

All primers and probes were designed to keep amplicons as short as possible using the CLC Workbench v20.0.4 (Qiagen, Hilden, Germany) and tested in the BiSearch primer-design algorithm [28]. The oligos were designed to specifically target bisulfite-converted DNA of the eight selected CpG islands and the PCa-specific methylated CpGs of the *DOCK2*, *FBXO30,* and *HAPLN3* regions described in Bjerre et al. [11]. All primers and probes encompassed CpG-dinucleotides, resulting in individual assays encompassing between 5 and 11 CpGs (S4 Table). Primers and probes were designed to target only methylated CpGs in hypermethylated CpG-islands and only unmethylated CpGs in hypomethylated CpG-islands.

Assays targeting the selected candidate CpG-islands were analyzed in a duplex with the *ALB* reference assay [29] on bisulfite-converted DNA templates extracted from whole blood and PNo and PCa tissue. The ddPCR reaction master mix was prepared as follows: 6 µl template, 10 pmol forward primer, 10 pmol reverse primer, 5 pmol methylation-specific FAM-labelled probe, 10 pmol VIC-labeled *ALB* probe (reference gene), 2xSupermix for probes no UTP (Bio-Rad) and molecular biology water (Sigma) to a final volume of 24 µl. Droplets were generated using the QX200 AutoDG Droplet Generator (Bio-Rad) and subsequent PCR amplification occurred in a Veriti Thermocycler (Applied Biosystems) at 95°C for 10 min and 45 cycles of 95°C for 15 sec, and 56°C for 1 min, finalizing at 98°C for 10 min. Amplified samples were stored at 4°C overnight before analysis on the QX200 reader (Bio-Rad).

Five methylation-specific assays *ACTRT2*, *EVX1–2*, *HOXD13*, *DOCK2,* and *HAPLN3,* and the *ALB* reference gene assay were used for multiplex construction (S4 Table). Each probe in the multiplex assay was labeled with different fluorophores and quenchers. ddPCR conditions were as described above, although the master mix contained primers and probes for all five methylation-specific assays and the *ALB* reference. The ddPCR data were generated using the QX600 six-color detection droplet reader (Bio-Rad) and analyzed using the QX Manager Standard Edition software (version 2.0.0, Bio-Rad). Bisulfite-converted methylated and unmethylated Human DNA templates as well as DNA from whole blood and no template controls were used for QC for each assay on each plate. Droplet counts > 15,000 were accepted.

### Specificity in clinical tissues and technical sensitivity

The ability of the mm-ddPCR assay to detect biomarkers specific to prostate tissue was validated on bisulfite-converted DNA extracted from whole blood, PNo and PCa tissue, and malignant colorectal, lung, breast, and ovarian tissue.

Genomic DNA purified from PCa tissue (used as a substitute for ctDNA from prostate cancer patients, which was not available) was used to evaluate assay linearity across all targeted methylation sites. A 7-point, 3-fold dilution series of bisulfite-converted DNA extracted from PCa tissue in 10 x diluted bisulfite-converted DNA extracted from whole blood with known concentration was used to test linearity. The dilution series was analyzed in triplicate for each assay in duplex with the *ALB* reference and in the multiplex. The slope and R^2^-value of the trend line were used for comparison of results. The lowest quantity detected by the multiplex assay using the dilution series was determined as the dilution in which all assays in triplicate successfully detected the biomarkers. This quantity was rounded to the nearest integer genomic copy number.

### Limit of blank (LOB) and clinical sensitivity in liquid biopsies from mCRPC patients

The LOB of the mm-ddPCR assay was determined by analyzing ccfDNA extracted from 4 ml plasma from men examined for PCa but with no histological findings of malignancy in the prostate. The test and validation cohorts were each constituted by 40 samples. The LOB of each assay in the mm-ddPCR was determined as the highest number of positive droplets measured in the non-cancer samples with a confidence level of 95%.

To analyze the clinical sensitivity, ccfDNA was purified from plasma obtained from a cohort of 20 mCRPC patients. Only 2 ml of plasma was available from each patient, from which ccfDNA was purified and analyzed by the mm-ddPCR assay, including quality control procedures as described. Results higher than the LOB were considered ctDNA-positive.

### Statistical analysis

The coefficient of determination reflecting the correlation between duplex and multiplex ddPCR assay measurements was calculated using linear regression according to the least squares method. All other statistical analyses were performed using R (v. 4.3.2). Wilcoxon Mann-Whitney tests were used to investigate differences between groups. The sensitivity of the multiplex ddPCR assay was estimated together with its corresponding 95% confidence intervals.

## Results

### Identification of methylation-specific candidate biomarkers

**Fig 1:**
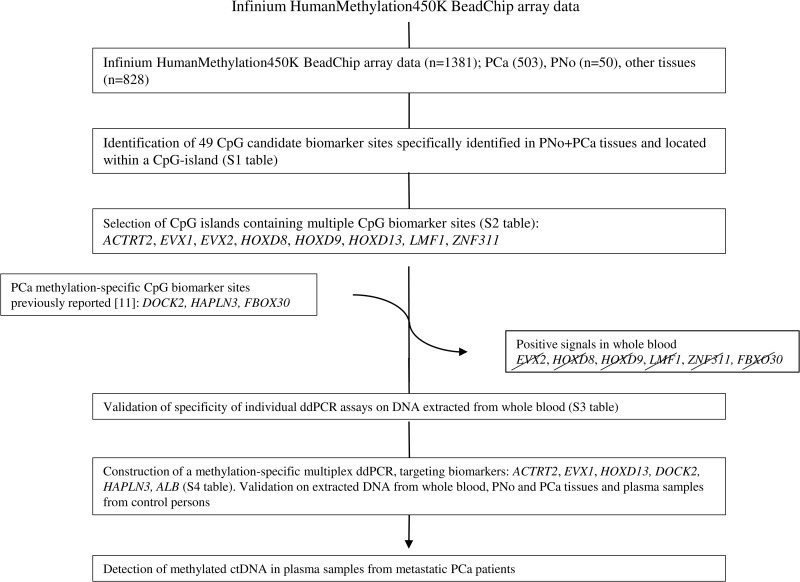
Flowchart illustrating biomarker selection and validation.

We took a stepwise approach by prioritizing CpGs that were either hypermethylated in prostate tissue and minimally methylated in other tissue or that exhibited minimal methylation in prostate tissue and hypermethylation in other tissues (Fig 1 and S1 Table). We narrowed down the selection to 49 top candidate CpG markers located within CpG islands (S2 Table). To identify regions showing a uniform prostate-specific methylation pattern, we focused on CpG islands containing multiple CpG units within a prostate methylation-specific signature. Based on these criteria, we identified nine candidate CpG islands located in the proximity of eight genes and containing two or more CpGs with a prostate tissue-specific methylation profile (S3 Table). We selected eight candidate CpG islands for further investigation, one for each gene.

Infinium Human Methylation450K BeadChip data were used to identify CpG-markers with unique methylation patterns in both PNo and PCa tissue. Candidate CpG islands in proximity of eight genes were selected due to the presence of multiple CpG candidates within the same CpG island. Validation of methylation-specific ddPCR assays targeting candidate regions in whole blood as well as PCa and PNo tissue identified three prostate-specific candidates: *ACTRT2*, *EVX1*, and *HOXD13*. Two assays targeting CpGs within *DOCK2* and *HAPLN3,* respectively, which were previously described to be methylated in PCa, were included in the multiplex assay.

### Biomarker candidate assay selection

Eight ddPCR assays were designed to target each of the selected CpG islands containing prostate methylation-conserved CpGs. Each assay was tested in conjunction with the *ALB* reference gene on extracted and bisulfite-converted DNA from whole blood, tissue from PNo and PCa, and plasma from non-PCa patients. We selected three assays that facilitated clear detection of three distinct genomic loci without any interference (S1 Fig). These assays were named after the nearest located genes *ACTRT2*, *EVX1,* and *HOXD13* (S3 Table). The CpG sites of *ACTRT2* were hypomethylated in prostate tissue whereas the CpG sites within *EVX1* and *HOXD13* were located in a hypermethylated CpG island. To enhance sensitivity, we included two PCa-specific targets in the mm-ddPCR assay. We designed ddPCR assays targeting *DOCK2*, *FBXO30* and *HAPLN3* CpGs previously reported to be specifically hypermethylated in PCa [11]. The *DOCK2* and *HAPLN3* assays were selected and included in the mm-ddPCR assay.

### Technical validation of sensitivity

The cluster position and background levels for all markers were checked to ensure avoidance of probe-primer cross-reactions from the included assays (S1 Fig). The technical sensitivity of the mm-ddPCR assay was evaluated using a 7-point 3-fold dilution series in a background of 1500 copies of bisulfite-converted genomic DNA copies from whole blood. All five selected assays displayed linear correlation when conducted in multiplex similarly to their performance in combination with the reference assay only (S2 Fig). All assays in the mm-ddPCR were able to detect eight copies of bisulfite-converted methylated DNA in all three replicates. The clinical limit of detection (LOD) will be measured in future studies using ctDNA positive plasma collected from PCa patients.

### Validation of specificity in clinical tissue

**Fig 2:**
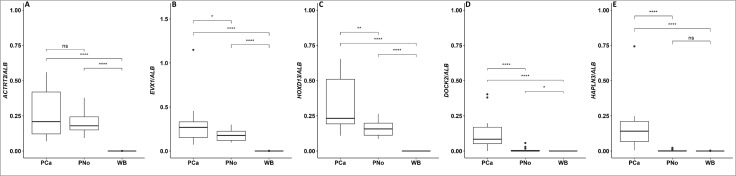
Levels of markers in whole blood, prostate normal (PNo) and cancer (PCa) tissues. Methylation-specific multiplex ddPCR was performed on DNA extracted from whole blood, PNo and PCa tissue. Results are shown in box-plots as the ratio of detected copies/µl of each prostate DNA biomarker relative to the number of reference *ALB* molecules. **(A)** *ACTRT2*, **(B)** *EVX1*, **(C)** *HOXD13*, **(D)** *DOCK2* and **(E)** *HAPLN3*. P-values were calculated using Mann-Whitney tests and are marked as follows: ns: p > 0.05; *: p ≤ 0.05; **: p ≤ 0.01; ***: p ≤ 0.001; ****: p ≤ 0.0001. WB: whole blood, PNo: normal prostate tissue, PCa: prostate cancer tissue.

To assess the specificity of the mm-ddPCR assay, we analyzed bisulfite-converted DNA extracted from tissue (PNo, PCa) and whole blood. Consistent with the specificity outlined in our bioinformatics analyses, the *ACTRT2*, *EVX1* and *HOXD13* assays detected significant amounts of biomarker levels in all tissue samples analyzed on DNA extracted from PCa and PNo but not on DNA from whole blood (Fig 2 and S3A Fig). In agreement with previous findings [11], *HAPLN3* and *DOCK2* assays detected a significant amount of biomarkers in DNA from all PCa samples and in 95% of cases, respectively, whereas only minimal levels were observed in DNA from PNo and none in whole blood.

We tested the specificity of the multiplex assay in cancer tissues other than PCa. The biomarkers were detected in some samples from various cancer types but not at a consistent level as seen for PNo and PCa tissue samples (S3 Fig). For instance in all CRC tissue, *EVX1*, *HOXD13,* and *DOCK2* were detected but not *ACTRT2* and *HAPLN3*. For other cancer types, the presence and concentration of biomarkers differed among the individual samples.

### Limit of blank (LOB) in non-PCa control plasma samples

**Fig 3:**
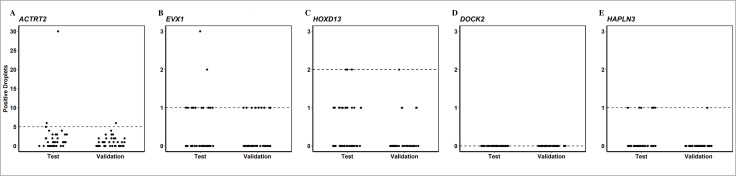
Limit of blank in plasma from control non-cancer patients. Analysis of 4 ml plasma from men examined for prostate cancer, but with no histological findings of malignancy in the prostate. Samples were divided into a test cohort (N = 40) and a validation cohort (N = 40). The dot plots show the number of positive droplets found for the test cohort (left) and the validation cohort (right) for each assay in the multiplex, **(A)** *ACTRT2*, **(B)** *EVX1*, **(C)** *HOXD13*, **(D)** *DOCK2,* and **(E)** *HAPLN3*. The dashed line indicates the limit of blank (LOB) for each biomarker assay as determined in the test cohort.

The LOB for each of the assays within the mm-ddPCR assay was established as described previously [30]. In order to use negative control samples closely resembling samples from PCa patients, we collected plasma from men examined for PCa in which no histological findings of malignancy were detected. Control samples were divided into a test cohort and a validation cohort. The test cohort was used to set the LOB, which was subsequently confirmed in the validation cohort. The bisulfite-converted concentration of ccfDNA in the test and validation cohort, respectively, was quantified as copies/ml using ddPCR (median = 841, range 392–5429 and median = 794, range 379–1861). In the test cohort, the 95% CI LOB was determined to be 5, 1, 2, 0, and 1 positive droplets for *ACTRT2*, *EVX1*, *HOXD13, DOCK2,* and *HAPLN3,* respectively (Fig 3). The validation cohort confirmed the LOB for all biomarkers in the multiplex. The individual biomarkers were considered positive, if the number of positive droplets exceeded the LOB. In one sample in the test cohort, the multiplex assay detected 30 positive droplets of *ACTRT2*, which is 5 times higher than the LOB. This sample showed signals below the LOB for the four other biomarkers.

### Clinical sensitivity in liquid biopsies from mCRPC patients

**Fig 4:**
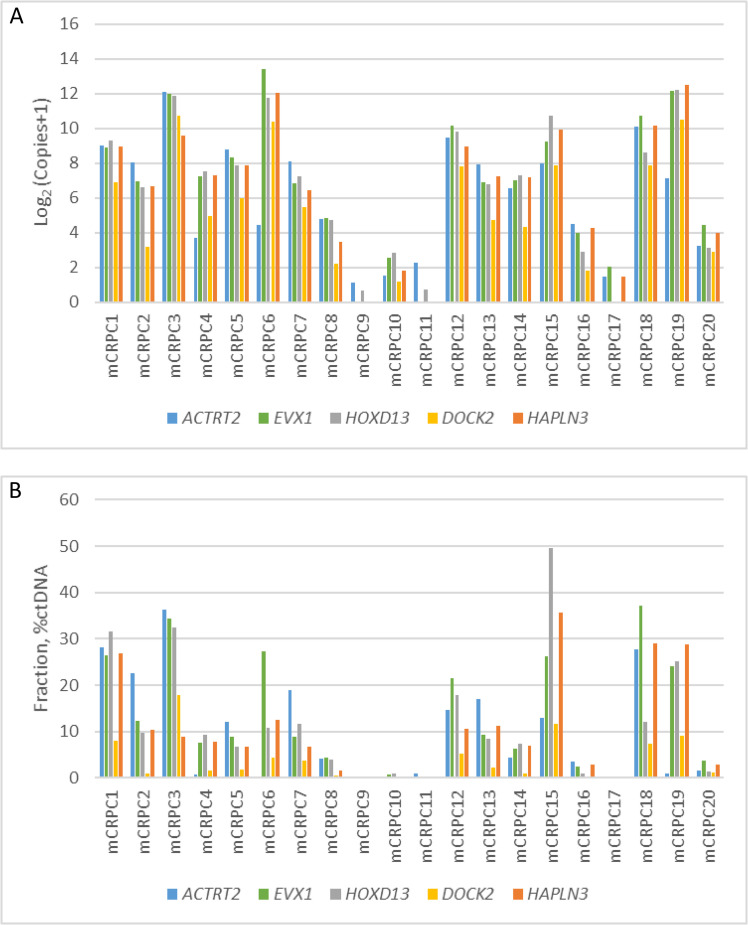
Levels of *ACTRT2*, *EVX1*, *HOXD13*, *DOCK2,* and *HAPLN3* biomarkers in plasma from metastatic castration-resistant prostate cancer (mCRPC) patients. **(A)** The log_2_ of the biomarker DNA copies +1 is shown for each biomarker in the multiplex. **(B)** The percentage of ctDNA detected. Blue = *ACTRT2,* green = *EVX1,* grey = *HOXD13*, Yellow = *DOCK2,* orange = *HAPLN3*.

To validate the mm-ddPCR as an assay for detection of ctDNA biomarkers in liquid biopsies from PCa patients, we used the assay to analyze plasma from 20 mCRPC patients where the median number of bisulfite-converted ccfDNA copies per ml plasma was 2201 (range 903–50671). Positivity for ctDNA was recorded when ctDNA copies exceeded the LOB previously determined. On this basis, the assay’s clinical sensitivity was 95% (95% CI, 85%−100%) with the multiplex assay successfully detecting biomarkers in 19 of 20 samples. In 80% of samples (16 of 20) positive results were demonstrated for all five biomarkers (Fig 4A). In four samples, less than five biomarkers were detected. One sample (mCRPC10) was positive for four biomarkers, one (mCRPC17) for *EVX1* and *HAPLN3*, and one sample (mCRPC11) was positive for *ACTRT2* only. The mCRPC9 sample was negative for all five markers. Interestingly, 19 samples were positive for at least one of the prostate-specific biomarkers, whereas 18 samples were positive for at least one of the PCa-specific biomarkers. Since the level of ctDNA was below the established LOD in the four samples not detected by all assays, future studies should involve larger cohorts to assess potential differences in the presence of the various biomarkers. In 80% of the mCRPC plasma samples, at least one biomarker showed a level of ctDNA > 2%. In 50% of the samples, the level of ctDNA in at least one biomarker was > 10% (Fig 4B). The highest level of ctDNA was in the mCRPC15 sample (*HOXD13* = 49.5%).

## Discussion

In this study, we identified three novel CpG-methylation patterns conserved in prostate tissue (*ACTRT2*, *EVX1,* and *HOXD13*). We successfully developed and validated an mm-ddPCR assay that detects ctDNA biomarkers containing the three methylation patterns together with two specific to PCa tissue (*DOCK2* and *HAPLN3*) in plasma from PCa patients within a single reaction tube. The mm-ddPCR assay detected methylated ctDNA biomarkers with a sensitivity of 95% (95% CI; 85%−100%) in mCRPC plasma samples and with ctDNA > 2% in 80% of the cases (Fig 4).

There is a large variation in the amount of detectable ctDNA in plasma from mCRPC patients. Results based on NGS technology have reported that ctDNA can be detected in 56–80% of mCRPC plasma samples [13–16]. A higher fraction of ctDNA-positive samples detected by the mm-ddPCR assay might be expected, as the ddPCR platform has been reported to be equal to or even more sensitive than NGS-based assays [17]. Additionally, the mm-ddPCR assay showed a specificity of 100% towards biomarkers in normal and cancerous prostate tissue samples (Fig 2) indicating that the assay can be used for universal PCa-derived ctDNA biomarker detection. The mm-ddPCR assay may therefore be a powerful alternative to NGS for simple, low-cost, high-throughput, universal, and very sensitive monitoring of mCRPC treatment response and progression. In addition, our multiplex assay may rapidly and concurrently estimate the fraction of ctDNA in mCRPC patients and indicate whether the patient would have sufficient material to perform ctDNA sequencing and obtain information on actionable alterations and the molecular mechanisms driving treatment response [13]. Future studies will address the functionality of the mm-ddPCR assay in larger cohorts.

We enhanced the sensitivity of the mm-ddPCR assay by designing a multiplex targeting five biomarkers within a single reaction tube. The assays for *ACTRT2*, *EVX1,* and *HOXD13* target methylation patterns conserved in prostate cells independently of the cancer status. Those for *HAPLN3* and *DOCK2* target methylation patterns specifically found in PCa cells [11]. DNA methylation biomarkers have been identified as tools for classifying mCRPC [31,32]. We found that prostate-conserved biomarkers were detected in 95% and PCa-specific biomarkers in 90% of the samples from mCRPC patients (Fig 4). To the best of our knowledge, this is the first study evaluating tissue-conserved biomarkers in PCa patients. Future studies of larger cohorts should address how these markers are detected in patients with different stages of PCa and how results associate with clinical parameters and survival.

The mm-ddPCR assay non-systematically detected biomarkers in tissue from other cancers than PCa (S3 Fig). The reason may be an aberrant methylation pattern frequently seen in cancer cells [33]. This could potentially cause a positive signal from a marker even in the absence of cancerous prostate cells. Such an event might explain the observation of a large number of positive *ACTRT2* droplets in one control plasma sample (Fig 3). The elevated number of *ACTRT2* droplets in this sample can most likely not be attributed to the patient having PCa. It cannot be ruled out, however, that this patient had a different type of cancer or a condition that could compromise the function of the prostate and thereby potentially cause secretion of prostate cfDNA into the blood. Consequently, the mm-ddPCR assay seems to provide a powerful tool for monitoring patients already diagnosed with PCa, but its application should be enhanced by incorporating additional clinical or biochemical markers for improved management of PCa.

Risk stratification and treatment guidance for PCa patients are based on several clinicopathological features such as PSA level, clinical stage, Gleason grade group, and histopathology [34]. Due to its low specificity, PSA is not always able to discriminate between benign and malignant prostate disease and a PSA result should be interpreted with caution [5,6]. High ctDNA levels at baseline and four weeks after treatment initiation are strongly associated with a poor response to first-line androgen receptor pathway inhibitor therapy and reduced overall survival in patients with mCRPC [15]. Therefore, ctDNA measurements may add significant value beyond PSA, and early detection of changes in ctDNA levels might be more efficient for the early identification of mCRPC patients likely to respond to the treatment. Moreover, in a disease characterized by significant bone involvement, defining radiological progression poses a consistent clinical challenge in the management of mCRPC. Consequently, a more reliable liquid biomarker could improve clinicians’ assessment of treatment, allowing for more timely decisions in the management plan.

PSA testing is simpler and low-priced compared to ctDNA measurements, but in certain situations PSA measurements do not necessarily reflect PCa progression, e.g., in patients with high-grade tumors and when the PSA production does not increase upon progression. Such patients could benefit from the enhanced sensitivity and specificity of ctDNA measurements offered by the mm-ddPCR assay.

## Conclusion

In summary, we have identified novel prostate specific CpG- methylation patterns and used them to develop a highly sensitive mm-ddPCR assay targeting ctDNA biomarkers in plasma from PCa patients. The mm-ddPCR assay has the potential to be a sensitive, fast, low-cost, and high-throughput tool for minimally invasive, blood-based treatment guidance and monitoring of disease progression in patients with advanced PCa. Future studies are needed to elucidate its functionality in other clinical contexts.

## Supporting information

S1 Table Samples included in the biomarker discovery analysis. (XLSX)

S2 Table Differentially methylated CpGs in prostate versus other tissues. (XLSX)

S3 Table Selected CpG islands. (XLSX)

S4 Table Primers and probes used in the multiplex ddPCR assay. (XLSX)

S1 Fig 1D and 2D QX Manager amplitude fluorescence plots of multiplex analysis of bisulfite converted DNA. (A) Examples of 1-D amplification fluorescence plots of each marker analyzed in duplicate: Hypermethylated human genomic control DNA (C05, C06), hypomethylated human genomic control DNA (D05, D06), two normal prostate tissue samples PNo10 (A01, A02) and PNo11 (B01, B02), and two prostate cancer tissue samples PCa21 (G09, G10) and PCa22 (H09, H10). Channel 1: FAM, *HOXD13*; channel 2: VIC, *ALB*; channel 3: Cy5.5, *EVX1*; channel 4: Cy5, *HAPLN3*; channel 5: ROX, *ACTRT2*; channel 6: ATTO590, *DOCK2*. Threshold lines are shown in pink. (B)-(E) 2-D amplification fluorescence plots. Clusters are defined on plots with identical coloring of droplets and name of target gene. *HOXD13 =* blue, *ALB =* green, *HAPLN3 =* red, *EVX1 =* purple, *ACTRT2 =* burgundy, and *DOCK2 =* light blue. (B) Hypermethylated human genomic control DNA, (C) Hypomethylated human genomic control DNA, (D) PCa21 + PCa22, (E) PNo10 + PNo11.
(PDF)

S2 Fig Performance of assays in duplex versus multiplex. A 7-point 3-fold dilution series of a tumor sample in a background of 1500 copies of bisulfite-converted DNA copies from whole blood was used to compare the sensitivity of each assay in duplex (with the reference gene) to that found by the multiplex. The correlation between measurements is indicated by the coefficients of determination (R^2^). (A) *ACTRT2*, (B) *EVX1*, (C) *HOXD13*, (D) *DOCK2,* (E) *HAPLN3*. The Y-axis displays the number of copies/µl detected using the multiplex assay and the X-axis the number of copies/µl detected using duplexes of the individual assays in combination with the ALB reference gene.
(PDF)

S3 Fig Levels of biomarkers in whole blood and tissue. Methylation-specific multiplex ddPCR was performed on bisulfite-converted DNA extracted from (A) whole blood (WB), normal prostate (PNo) and prostate cancer (PCa) tissue, (B) colorectal cancer (CRC), lung cancer (LC), breast cancer (BC) and ovarian cancer (OC) tissue. Results are shown as the ratio of copies/ml between each prostate DNA biomarker and the reference *ALB* gene. The results of the individual markers in the multiplex are colored: *ACTRT2 =* blue, *EVX1 =* green, *HOXD13 =* grey, *DOCK2 =* yellow, *HAPLN3 =* orange.
(PDF)
