# Supplementary figures and images for: Methylation-specific, universal, and highly sensitive identification of ctDNA in prostate cancer by multiplex droplet digital PCR
Source: PLoS One. 2026 Jan 29;21(1):e0340786. doi: 10.1371/journal.pone.0340786 (PMC12854444; doi:10.1371/journal.pone.0340786)

S1 Fig.

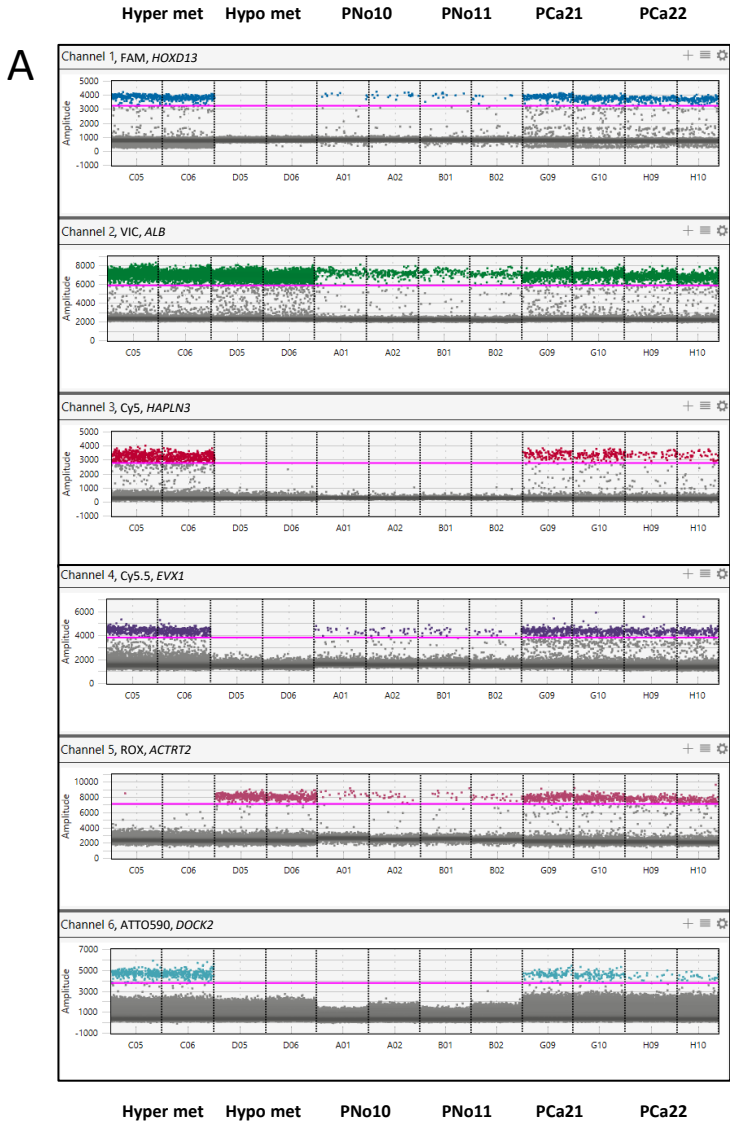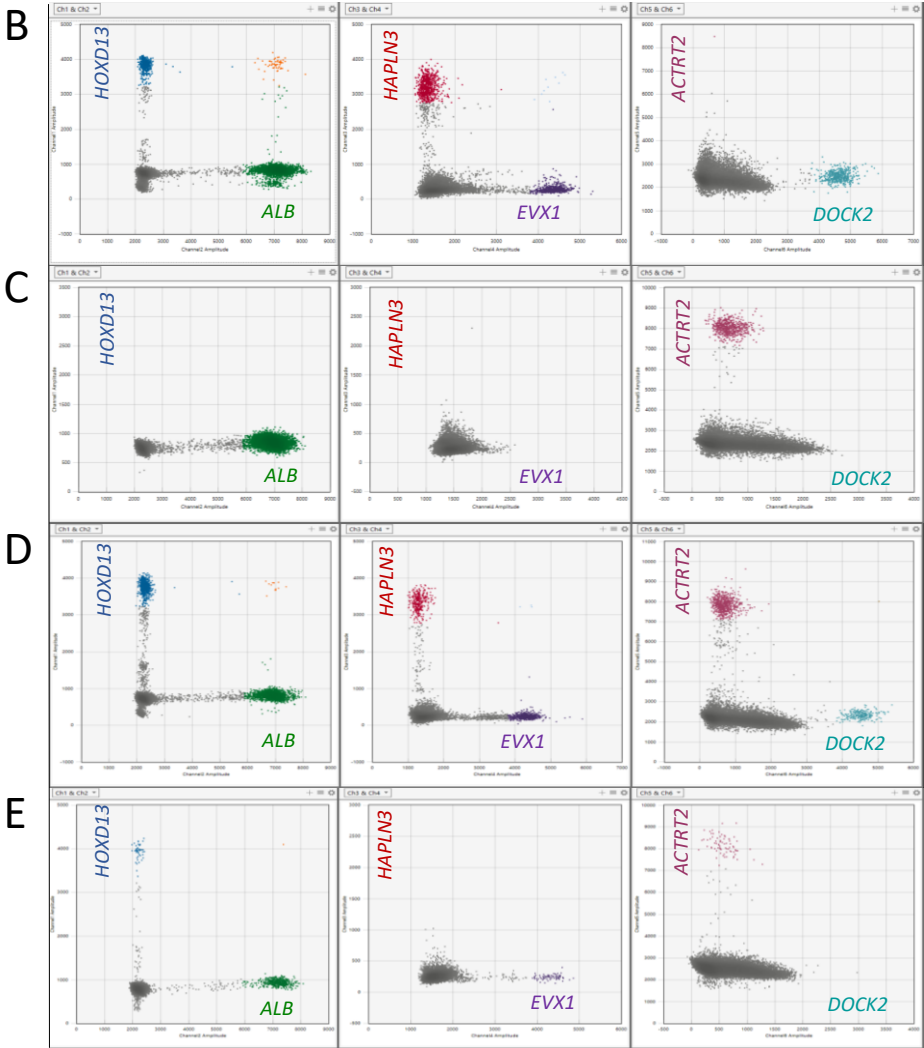

Supplement: S1 Fig — (A) Examples of 1-D amplification fluorescence plots of each marker analyzed in duplicate: Hypermethylated human genomic control DNA (C05, C06), hypomethylated human genomic control DNA (D05, D06), two normal prostate tissue samples PNo10 (A01, A02) and PNo11 (B01, B02), and two prostate cancer tissue samples PCa21 (G09, G10) and PCa22 (H09, H10). Channel 1: FAM, HOXD13; channel 2: VIC, ALB; channel 3: Cy5.5, EVX1; channel 4: Cy5, HAPLN3; channel 5: ROX, ACTRT2; channel 6: ATTO590, DOCK2. Threshold lines are shown in pink. (B)-(E) 2-D amplification fluorescence plots. Clusters are defined on plots with identical coloring of droplets and name of target gene. HOXD13 = blue, ALB = green, HAPLN3 = red, EVX1 = purple, ACTRT2 = burgundy, and DOCK2 = light blue. (B) Hypermethylated human genomic control DNA, (C) Hypomethylated human genomic control DNA, (D) PCa21 + PCa22, (E) PNo10 + PNo11. (PDF) [file pone.0340786.s005.pdf]

A

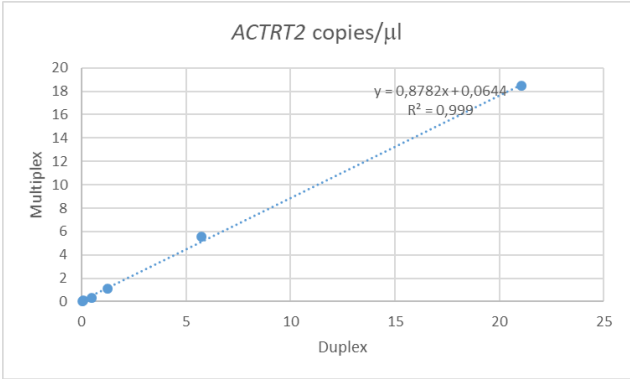

D

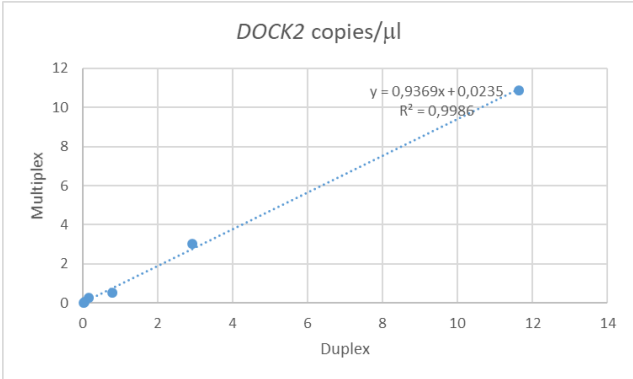

B

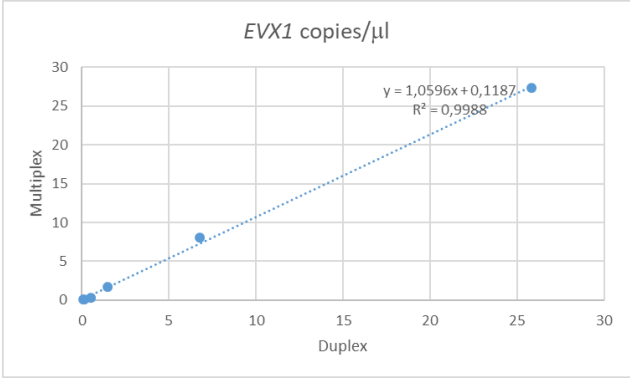

E

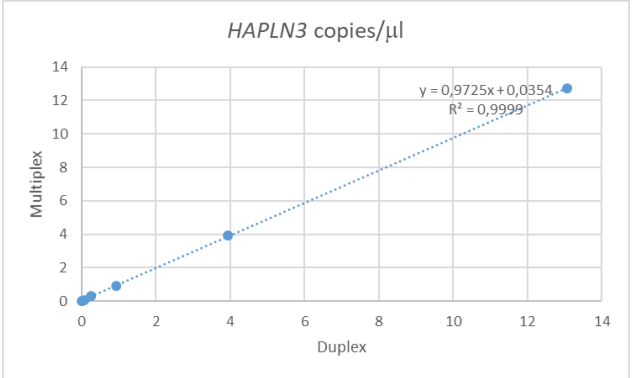

C

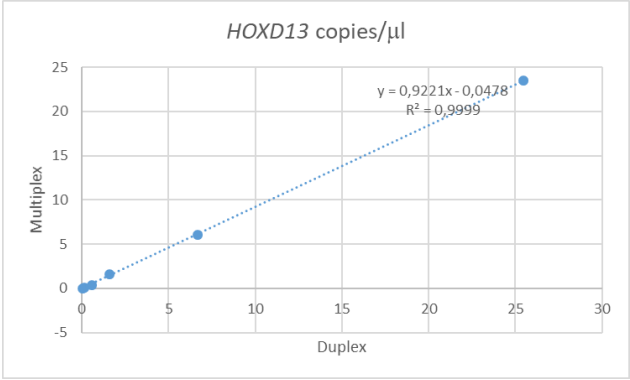

Supplement: S2 Fig — A 7-point 3-fold dilution series of a tumor sample in a background of 1500 copies of bisulfite-converted DNA copies from whole blood was used to compare the sensitivity of each assay in duplex (with the reference gene) to that found by the multiplex. The correlation between measurements is indicated by the coefficients of determination (R2). (A) ACTRT2, (B) EVX1, (C) HOXD13, (D) DOCK2, (E) HAPLN3. The Y-axis displays the number of copies/µl detected using the multiplex assay and the X-axis the number of copies/µl detected using duplexes of the individual assays in combination with the ALB reference gene. (PDF) [file pone.0340786.s006.pdf]

A

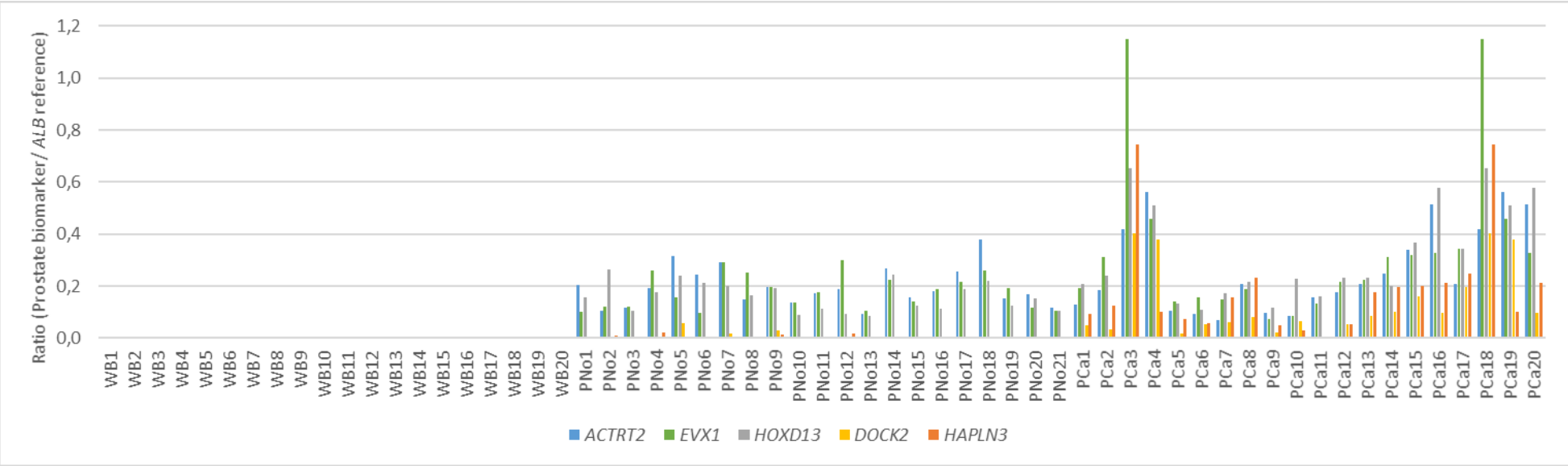

B

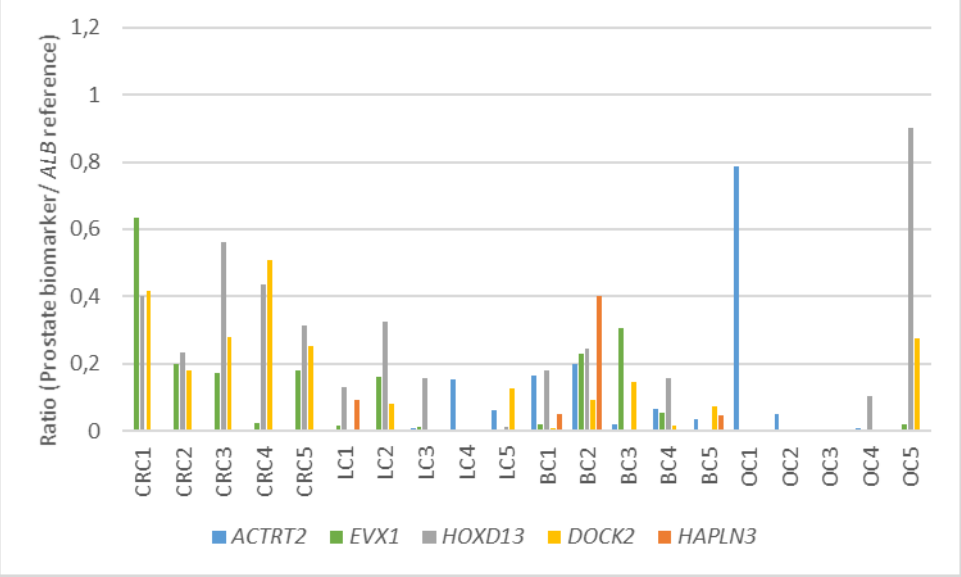

Supplement: S3 Fig — Methylation-specific multiplex ddPCR was performed on bisulfite-converted DNA extracted from (A) whole blood (WB), normal prostate (PNo) and prostate cancer (PCa) tissue, (B) colorectal cancer (CRC), lung cancer (LC), breast cancer (BC) and ovarian cancer (OC) tissue. Results are shown as the ratio of copies/ml between each prostate DNA biomarker and the reference ALB gene. The results of the individual markers in the multiplex are colored: ACTRT2 = blue, EVX1 = green, HOXD13 = grey, DOCK2 = yellow, HAPLN3 = orange. (PDF) [file pone.0340786.s007.pdf]
